# The Effect of Phenoloxidase Activity on Survival Is Host Plant Dependent in Virus-Infected Caterpillars

**DOI:** 10.1093/jisesa/ieaa116

**Published:** 2020-10-22

**Authors:** Justine L Resnik, Angela M Smilanich

**Affiliations:** 1 Department of Biochemistry, University of Nevada, Reno, NV; 2 Department of Biology, University of Nevada, Reno, NV

**Keywords:** Junonia coenia densovirus, fitness, immune response, pathogen, host plant

## Abstract

An important goal of disease ecology is to understand trophic interactions influencing the host–pathogen relationship. This study focused on the effects of diet and immunity on the outcome of viral infection for the polyphagous butterfly, *Vanessa cardui* Linnaeus (Lepidoptera: Nymphalidae) (painted lady). Specifically, we aimed to understand the role that larval host plants play when fighting a viral pathogen. Larvae were orally inoculated with the entomopathogenic virus, Junonia coenia densovirus (JcDV) (Family *Parvoviridae*, subfamily *Densovirinae*, genus *Protoambidensovirus*, species *Lepidopteran protoambidensovirus 1*) and reared on two different host plants (*Lupinus albifrons* Bentham (Fabales: Fabaceae) or *Plantago lanceolata* Linnaeus (Lamiales: Plantaginaceae)). Following viral infection, the immune response (i.e., phenoloxidase [PO] activity), survival to adulthood, and viral load were measured for individuals on each host plant. We found that the interaction between the immune response and survival of the viral infection was host plant dependent. The likelihood of survival was lowest for infected larvae exhibiting suppressed PO activity and feeding on *P. lanceolata*, providing some evidence that PO activity may be an important defense against viral infection. However, for individuals reared on *L. albifrons*, the viral infection had a negligible effect on the immune response, and these individuals also had higher survival and lower viral load when infected with the pathogen compared to the controls. Therefore, we suggest that host plant modifies the effects of JcDV infection and influences caterpillars’ response when infected with the virus. Overall, we conclude that the outcome of viral infection is highly dependent upon diet, and that certain host plants can provide protection from pathogens regardless of immunity.

A primary goal of insect disease ecology is to understand how interactions with the environment influence host–pathogen dynamics (Keesing et al. 2010). Within this framework, ecologists can investigate whether the host’s diet mediates the outcome of the infection, and in the case of herbivorous insects, whether different plants offer refuge from pathogens and parasites via immune enhancement (Shikano et al. 2010, Hansen et al. 2017, Slinn et al. 2018), chemical suppression (Singer et al. 2009, Sternberg et al. 2012, del Campo et al. 2013), or a combination of both immune enhancement and chemical suppression (Barthel et al. 2016). Jeffries and Lawton (1984) defined the term ‘enemy-free space’ as the situation, where an organism is better protected from a natural enemy in one environment versus an alternative environment. For herbivorous insects, certain host plants can provide ‘enemy-free space’ by enhancing defenses against pathogens and parasites, thus increasing overall fitness, and leading to a selective advantage in the ‘enemy-free space’ environment (Murphy 2004, Viney et al. 2005). In this study, we investigated whether alternative host plant environments affect the immunocompetence and survival of painted lady caterpillars (*Vanessa cardui*: Nymphalidae) experimentally infected with the entomopathogenic virus, Junonia coenia densovirus (JcDV) (Family *Parvoviridae*, subfamily *Densovirinae*, genus *Protoambidensovirus*, species *Lepidopteran protoambidensovirus 1*).

Previous studies investigating the tri-trophic interactions between diet, herbivores, and pathogens, have demonstrated that resistance to pathogens can be influenced by host plant traits (Raymond et al. 2002, Cory and Myers 2004, Cory and Hoover 2006, Shikano et al. 2010, Smilanich et al. 2018). Even though studies have linked plant traits such as nutritional quality and secondary metabolite composition to immune strength (reviewed in Ponton et al. 2013), only a limited number of cases show the basis of disease resistance can be directly linked to modification of immune parameters by dietary nutrients (Lee et al. 2006, Ponton et al. 2020) and secondary metabolites (del Campo et al. 2013, Barthel et al. 2016). Moreover, the link between certain immune parameters (i.e., phenoloxidase [PO]) and surviving infection is not always positive, with several studies showing no effect of the immune response on measures of disease resistance (Saejeng et al. 2010, Myers et al. 2011, Scholefield et al. 2019). Recent evidence from monarch caterpillars (*Danaus plexippus*) suggests that host plant chemistry can play a critical role in surviving an infection of the protozoan parasite *Ophryocystis elektroscirrha* (OE). Monarch caterpillars reared on host plant species with high cardenolide concentrations downregulated a handful of immune genes, but were more likely to survive an infection, suggesting a key role of plant chemistry in protection and mediation of the host–pathogen interaction (Smilanich and Nuss 2019, Tan et al. 2019). Thus, the role of the immune system in protection against pathogens may be heavily influenced by host plant identity. We sought to further understand the effect of host plant on immune strength and the role that immunity plays in survival after an infection challenge.

## Methods and Materials

Using *V. cardui* eggs obtained commercially (Carolina Biological), we conducted an experiment Spring 2017 in Reno, NV. We used two host plants: a non-native (*Plantago lanceolata*: Plantaginaceae) and a native host plant (*Lupinus albifrons*: Fabaceae). Larvae were orally inoculated with JcDV, a nonenveloped, single-stranded DNA virus first isolated from the buckeye caterpillar (*Junonia coenia*) (Rivers and Longworth 1972, Mutuel et al. 2010), but not restricted to this specific host (Smilanich et al. 2018). *Vanessa cardui* is a permissive host, and although widespread prevalence of JcDV in wild populations of *V. cardui* is currently unknown, there is evidence that the virus is present in field collected adults (Smilanich et al., unpublished data). We compared the effects of the virus on *V. cardui* immunity and survival to adulthood when larvae were reared on the two host plants. For more detail on the experimental design and analysis, see Supp Information [online only].

## Results and Discussion

**Fig. 1.**
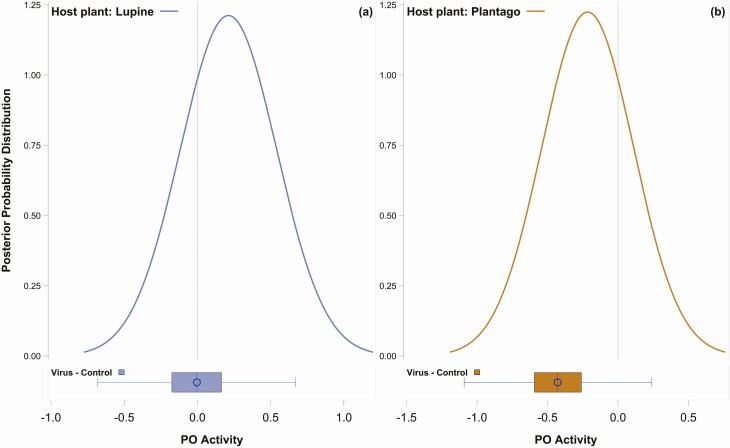
Panels (a) and (b) show posterior probability plots depicting the main effect of host plant on PO activity. The distribution of PO activity for the host plant, *L. albifrons,* is in the left panel and *P. lanceolata* in the right panel. The vertical line within the plot shows a zero effect size, or no difference between groups (see Supp Table 1 [online only] for unscaled mean PO values). Boxplots beneath posterior distributions show the interaction between host plant and virus on PO activity. Negative values indicate that the predictor variable decreased PO activity and vice versa for positive values. Thus, in the case of Plantago × Virus (right panel), there is a negative effect of virus on PO activity, while the Lupine × Virus interaction (left panel) shows PO values close to zero, or no difference between virus and control groups.

We found that the effect of viral infection on the immune response changed depending upon the host plant consumed, showing a strong interaction between viral infection and host plant consumed. First, as shown in the posterior probability distributions, the main effect of host plant on PO activity was close to zero (posterior probability mean [β] = 0.2327, highest posterior density interval [HPDI] = −0.1235 to 0.3537), indicating that there was little difference in the effect between *L. albifrons* and *P. lanceolata* on PO activity (Fig. 1a and b; see Supp Table 1 [online only] for unscaled data with means and SD). However, the effect sizes on PO activity changed dramatically when viral infection was considered, and showed a similar pattern for each plant species, however, the magnitude of the effect was larger on *P. lanceolata*. Specifically, when caterpillars feeding on *L. albifrons* were infected with JcDV, their PO activity was suppressed compared to control conditions, but the effect size was close to zero (β: virus – control = −0.0030, HPDI = −0.4718 to 0.5713, Fig. 1a). The pattern for caterpillars feeding on *P. lanceolata* was similar, but the difference in PO activity between virus and control individuals was larger (β: virus – control = −0.4277, HPDI = −0.9239 to 0.0454, Fig. 1b). Thus, the results for the immune response show that pathogen effects on the caterpillar host are not static but can change according to the host’s interaction with the environment (Smilanich et al. 2009, Cotter et al. 2011, Singer et al. 2014, Slinn et al. 2018), which in this case is the plant species consumed.

**Fig. 2.**
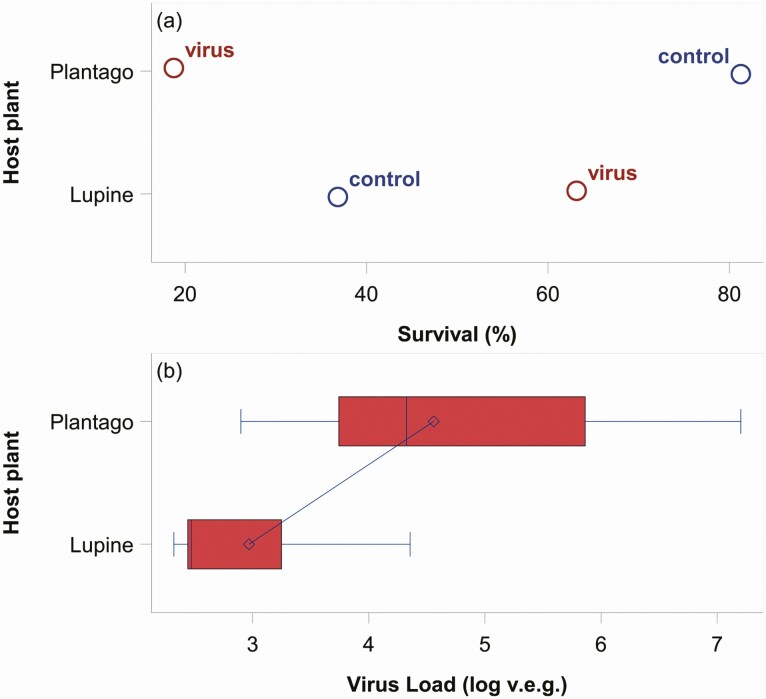
Top panel (a) shows differences in survival frequency for *V. cardui* caterpillars on each host plant under infected (virus) and uninfected (control) conditions. Notably, survival was higher for control caterpillars on Plantago while virus-infected caterpillars had higher survival on Lupine. The bottom panel (b) shows virus load of caterpillars reared on each host plant (expressed as log-transformed viral equivalent genomes). Individuals reared on Lupine had lower viral loads compared to individuals reared on Plantago, putatively explaining differences in survival frequency.

The outcome of the differing PO activity was reflected in the survival data, but only for *P. lanceolata*. The parsimonious logit model showed that survival was best predicted by the interaction between host plant and infection status (parameter estimate = 0.2991, *χ*^2^ = 9.91, *P* = 0.0016), where the likelihood of survival was higher on *L. albifrons*, but only under infection conditions (Fig. 2a; see Table 1 for model parameter estimates). When larvae were uninfected, the likelihood of survival was higher on *P. lanceolata*. In addition to the survival data, we found that within the infection treatment, mean viral load was lower in larvae reared on *L. albifrons* compared to *P. lanceolata* (β: *L. albifrons* – *P. lanceolata* = −1.590, HPDI = −2.7454 to −0.5169, Fig. 2b). We can putatively attribute the lower survival in infected *P. lanceolata*-reared individuals to the suppressed immune response during infection leading to the higher viral loads. This result was not shared with the native host plant, *L. albifrons*, suggesting that not only was PO activity not playing a protective role, but also that it was not vital since survival was actually higher and viral loads lower. Thus, the effectiveness of PO against pathogens appears to be heavily influenced by environment (i.e., host plant), perhaps explaining why several studies have found it to be a poor predictor of disease resistance (Saejeng et al. 2010, Myers et al. 2011, Scholefield et al. 2019).

**Table 1.** Model structures for the saturated and parsimonious logit models analyzing the effect of host plant (*P. lanceolata* or *L. albifrons*) and virus (yes or no) on caterpillar survival to adulthood (yes or no)

Model parameters	Saturated model	SE	χ ^2^	*P* value
	Estimate			
Host plant	0.1162	0.1071	1.18	0.2778
Virus	0.0213	0.1060	0.04	0.8605
Survival	0.4540	0.1019	19.84	<0.0001
Host plant × survival	−0.0260	0.1054	0.06	0.8049
Virus × survival	−0.1655	0.1072	2.38	0.1225
Host plant × virus × survival	0.3145	0.0972	10.47	0.0012
Likelihood ratio			1.29	0.2565
Model parameters	Parsimonious model	SE	χ ^2^	*P* value
	Estimate			
Host plant	0.0639	0.0918	0.49	0.4860
Virus	−0.0492	0.0917	0.29	0.5919
Survival	0.4504	0.1003	20.18	<0.0001
Host plant × virus × survival	0.2991	0.0950	9.91	0.0016
Likelihood ratio			3.71	0.2928

In both models, the interaction between host plant and virus are significant predictors of survival. See main text for specific patterns.

We conclude that the environment under which infection occurs is an important determinant in infection outcome on the host (Graham et al. 2011). In this study, the effects of viral infection led to suppressed immunity, lower survival, and higher viral loads, but only when larvae were reared on one of the host plants (*P. lanceolata*). For individuals reared on *L. albifrons*, survival was enhanced when infected with the virus, regardless of immunocompetence as measured by PO. This curious result requires further investigation, but it is noteworthy that at least one other study found higher survival frequency in individuals infected with a densovirus (Xu et al. 2014), indicating that under certain conditions not all viruses are pathogenic (Suttle 2013, Roossinck 2015, Xu et al. 2020). We suggest that future studies delve into differences in host plant traits that may contribute to different outcomes of pathogen infection. The aforementioned exploration of plant chemistry is a worthwhile follow-up for this study and similar studies interested in host plant–based variation in disease resistance. Not only could secondary metabolites interact with viral particles at the midgut-hemocoel interface (Hoover et al. 2000), but also at the phylloplane where surface chemistry could interact with virions to interfere with infection (Stevenson et al. 2010).

## Supplementary Material

ieaa116_suppl_Supplementary_Material
