# Corrigendum to: The Effect of Phenoloxidase Activity on Survival Is Host Plant Dependent in Virus-Infected Caterpillars

**DOI:** 10.1093/jisesa/ieab060

**Published:** 2021-11-02

**Authors:** Justine L Resnik, Angela M Smilanich

**Affiliations:** 1 Department of Biochemistry, University of Nevada, 1664 N. Virginia St., Reno, NV 89557, USA; 2 Department of Biology, University of Nevada, 1664 N. Virginia St., Reno, NV 89557, USA

Correction of “Justine L. Resnik and Angela M. Smilanich. 2020. The Effect of Phenoloxidase Activity on Survival Is Host Plant Dependent in Virus-Infected Caterpillars. J. Insect Sci.”

DOI: 10.1093/jisesa/ieaa116

In the published manuscript, the taxonomic name of the virus was misspelled. The authors have corrected the misspelling of “*Parvovirididae: Densovirinae, Lepidopteran Potoambidensovirus 1*” to read: “Family *Parvoviridae*, subfamily *Densovirinae*, genus *Protoambidensovirus*, species *Lepidopteran protoambidensovirus 1*”.

