# Supplementary material for: The Effect of Phenoloxidase Activity on Survival Is Host Plant Dependent in Virus-Infected Caterpillars
Source: J Insect Sci. 2020 Oct 22;20(5):26. doi: 10.1093/jisesa/ieaa116 (PMC7583276; doi:10.1093/jisesa/ieaa116)
Supplement: ieaa116_suppl_Supplementary_Material [file ieaa116_suppl_supplementary_material.docx]

**The effect of phenoloxidase activity on survival is host plant dependent in virus-infected caterpillars**

Justine L. Resnik^1^ and Angela M. Smilanich^2^

^1^*Department of Biochemistry, 1664 N. Virginia St. University of Nevada, Reno, NV 89557 USA*

^2^*Department of Biology, 1664 N. Virginia St. University of Nevada, Reno, NV 89557 USA*

**Corresponding Author:**

Angela Smilanich

Department of Biology

1664 N. Virginia St.

University of Nevada, Reno, NV 89557 USA

Email: [asmilanich@unr.edu](mailto:asmilanich@unr.edu)

Ph: 775-682-7756

**Experimental Design**

*Vanessa cardui* eggs obtained from Carolina Biological (N. Carolina U.S.A.) were reared in an incubator with a photoperiod of 16:8 L:D with a 25˚C daytime temperature and a 20˚C nighttime temperature. Caterpillars were selected at random to be reared on either *Plantago lanceolata* or *Lupinus albifrons*. *Plantago lanceolata* was collected from Idlewilde Park in Reno, NV. *Lupinus albifrons* was collected in Humboldt-Toiyabe National Forest near Verdi, CA. Caterpillars were reared individually on the designated host plant upon hatching in 2 oz. disposable soufflé cups (Comfy Package). Approximately half of the caterpillars from each plant group were then randomly chosen to be infected with JcDV at a concentration of 1 x 10^6^ viral particle per μl. This is a modified concentration based upon the LD_50_ found for buckeye larvae (Smilanich et al. 2018). All larvae were weighed on the first day of their 5^th^ instar. Caterpillars chosen to be infected were inoculated on the 1st day of their 5th instar (see Virus Inoculation). JcDV used for inoculations was sent to our lab after being isolated and purified in the M. Ogliastro Lab, University of Montpellier, Montpellier, France (Mutuel et al. 2010). For further information on virus purification and isolation see Mutuel et al. 2010. Four days post-infection, hemolymph was collected to assess the phenoloxidase activity (see Immune Assay). Caterpillars were then reared to adulthood to assess for survival. All individuals who emerged from their pupae were recorded as individuals who survived.

**Virus Inoculation**

Newly molted 5th instar *V. cardui* larvae were inoculated with JcDV at a concentration of 1 x10^6^ viral particles per µl. The larvae were inoculated with JcDV by placing 1 µl of JcDV suspension on a 10mm leaf disk obtained using a hole puncher. A virus coated leaf disk was placed in an empty 1 oz plastic soufflé cup with one individual. To ensure that the leaf disk was consumed, and the caterpillars were inoculated, the individuals were restricted from additional food for 24 hours. Following inoculation, caterpillars were returned to their individual 2 oz. soufflé cups.

**Immune Assay**

A colorimetric assay was used to measure phenoloxidase (PO) activity in 5th instar caterpillars (Adamo 2004). Phenoloxidase is responsible for converting phenols into melanin during melanogenesis (Gonzalez-Santoyo and Cordoba-Aguilar 2012). This melanin can then be used for melanization: which is the process of depositing melanin around infected tissue (Gonzalez-Santoyo and Cordoba-Aguilar 2012). Phenoloxidase can be found either in an active state in hemolymph or in a stored state inside hemocytes. Here we chose to measure the active state of the enzyme since caterpillars were actively infected. Hemolymph samples were collected from larvae 4 days post infection (or 4^th^ day of the fifth instar for controls). We chose 4 d.p.i. as this time period has been shown to be the phase of the infection when viral replication at peak (Mutuel et al. 2010). Hemolymph was collected by gently piercing the cuticle of the A1 abdominal segment with a hand-pulled Pasteur pipette needle (Smilanich et al. 2009). Caterpillars were then squeezed until a total of 10 µl was collected using a micropipette. A total of 10 µl was added to 500 µl of ice-cold phosphate buffered saline (Millipore Sigma) in a 1.5 mL Eppendorf tube. All solutions and supplies (including pipette tips) were kept on ice during this process to prevent enzyme degradation. Once all samples were prepped, they were vortexed and 100 µl of each sample was loaded into a 96 well plate in duplicate. 200 µl of L-DOPA (0.118g L-DOPA in 30ml deionized water) (Millipore Sigma) was added to each well and the reading was recorded immediately using an iMark Microplate Absorbance Reader (Bio-Rad). The absorbance was read at 490 nm every 30s for 45m. The slope of the resulting line was used to analyze PO activity. For analyses, only the linear portion of the curve was utilized. All samples were linear until 30min, thus the data were truncated at 30min.

**Viral Detection**

All larvae (including controls) were screened for virus load using qPCR analysis. The qPCR was performed at the end of the experiment after all individuals had died. Viral load was only performed on larval that died during the experiment. Adults were not screened for virus. Samples were stored at 4 degrees C until tissue extraction. Insects were homogenized using a TissueLyser II with a 3 mm tungsten carbide bead (Qiagen, Hilden, North Rhine-Westphalia, Germany). Total DNA extraction was performed with 20 mg of the homogenized tissue from an individual caterpillar using a Qiagen DNeasy Blood and Tissue Kit (Purification of Total DNA from Insects using the DNeasy Blood and Tissue Kit, Qiagen #69506) (Wang et al. 2013). All of the samples were normalized to 10 ng/µl of DNA and qPCR was performed using 0.2µM each of JcDV specific forward and reverse primers for the VP4 gene (Wang et al. 2013) and 5 µl iTaq Universal SYBR Green Supermix in a total volume of 7 µl. Reactions were run using a BioRad CFX96 Thermal Cycler with an initial denaturing step at 95**°**C for 5 minutes followed by 45 cycles under the following parameters: 95**°**C for 10 seconds, 60**°**C for 15 seconds, 72**°**C for 15 seconds. A melt curve was run following amplification, the JcDV virus stock solution (provided by M. Ogliastro, INRA-University of Montpellier, France) was serially diluted and used to make a standard curve with a linear range over seven orders of magnitude (1.0 x 10^3^ to 1.0 x 10^9^ gene copies/µl). Viral load was calculated as absolute quantification of gene copy number using the threshold cycle (C_t_) protocol as outlined in Schmittgen and Livak (2008) (Schmittgen and Livak 2008). Samples were run in duplicate and averaged. Using this standard curve, mean C_t_ values for VP4 were used to calculate the concentration of JcDV in each sample. Based on this standard curve, we extrapolated the absolute quantity of viral equivalent genomes (v.e.g.) in each sample (N = 19, *P. lanceolata*; N = 5, *L. albifrons*).

**Statistical Analysis**

All statistical analyses were performed in SAS (Statistical Analysis Software v.9.4, Cary Institute N.C., USA). Data from the immune assays were analyzed using Bayesian regression models with the genmod procedure (PROC GENMOD) in SAS. The model included the main effects of hostplant and virus as well as the interaction between the two predictor variables on phenoloxidase (PO) activity. The values for PO activity were scaled to z scores prior to running the model. The model had a burn-in size = 2000, MC sample size = 10000, and a normal prior distribution (mean = 0, SD = 10^6^). To summarize the output of the model, we used the posterior probability means (β) for each comparison (hostplant, virus, and the interaction) and the 95% highest posterior density interval (HPDI) (McElreath 2020). For figures summarizing this model, the x-axis displays the effect size (difference in means) of PO activity for each predictor variable and the interaction between the two predictor variables. Negative effect size values indicate that the predictor variable decreased PO activity and vice versa for positive values. The HPDI shows the narrowest portion of the posterior probability distribution corresponding to 95% of PO values in the distribution (McElreath 2020). Thus in the case of hostplant effect on PO activity the highest density of PO values are close to zero. To analyze the effect of hostplant and virus infection on survival, we used a logit model (PROC CATMOD), including hostplant (*P. lanceolata* or *L. albifrons*) and infection status (virus, control) as predictor variables and survival (yes, no) as the response. We first analyzed the data with a saturated model that included all interactions and their main effects (Table 2). Interactions that were not significant were dropped from the model (Host x Survival and Virus x Survival), but main effects and the three-way interaction (Host x Virus x Survival) were kept. The reported model is the parsimonious model that fit the data.

**References**

Adamo, S.A. (2004) Estimating disease resistance in insects: phenoloxidase and lysozyme-like activity and disease resistance in the cricket Gryllus texensis. *Journal of Insect Physiology,* **50**, 209-216.

Gonzalez-Santoyo, I. and Cordoba-Aguilar, A. (2012) Phenoloxidase: a key component of the insect immune system. *Entomologia Experimentalis Et Applicata,* **142**, 1-16.

McElreath R (2020) Statistical rethinking. CRC Press, Boca Raton, FL.

Mutuel, D., Rayallec, M., Chabi, B., Multeau, C., Salmon, J.M., Fournier, P. and Ogliastro, M. (2010) Pathogenesis of Junonia coenia densovirus in Spodoptera frugiperda: A route of infection that leads to hypoxia. *Virology,* **403**, 137-144.

Schmittgen, T.D. and Livak, K.J. (2008) Analyzing real-time PCR data by the comparative C-T method. *Nature Protocols,* **3**, 1101-1108.

Smilanich, A.M., Dyer, L.A., Chambers, J.Q. and Bowers, M.D. (2009) Immunological cost of chemical defence and the evolution of herbivore diet breadth. *Ecology Letters,* **12**, 612-621.

Smilanich, A.M., Langus, T.C., Doan, L., Dyer, L.A., Harrison, J.G., Hsueh, J. and Teglas, M.B. (2018) Host plant associated enhancement of immunity and survival in virus infected caterpillars. *Journal of Invertebrate Pathology,* **151**, 102-112.

Wang, Y., Grenet, A.S.G., Castelli, I., Cermenati, G., Ravallec, M., Fiandra, L., Debaisieux, S., Multeau, C., Lautredou, N., Dupressoir, T., Li, Y., Casartelli, M. and Ogliastro, M. (2013) Densovirus Crosses the Insect Midgut by Transcytosis and Disturbs the Epithelial Barrier Function. *Journal of Virology,* **87**, 12380-12391.

Table 1. Phenoloxidase activity (PO) mean, standard deviation (SD), and sample size (N) for *V. cardui* larvae reared on each host plant and under either viral or control conditions. PO data are unscaled and depict the average optical density per minute.

|  | **PO Activity** | | |
| --- | --- | --- | --- |
| **Host plant** | **Mean** | **SD** | **N** |
| *Lupinus albifrons* | 47.24 | 36.48 | 64 |
| Virus | 42.40 | 36.14 | 32 |
| Control | 52.09 | 36.75 | 32 |
| *Plantago lanceolata* | 41.63 | 45.33 | 57 |
| Virus | 22.34 | 38.49 | 31 |
| Control | 64.64 | 45.49 | 26 |
| **Virus** |  |  |  |
| Control | 57.71 | 39.62 | 58 |
| Virus | 32.53 | 38.37 | 63 |
